# Insights from a cross-sectional survey of neonatal resuscitation instructors from India

**DOI:** 10.1038/s41598-023-42382-w

**Published:** 2023-09-14

**Authors:** Purvi Patel, Somashekhar Nimbalkar, Mayur Shinde

**Affiliations:** 1 Department of Pediatrics, Pramukhswami Medical College, Bhaikaka University, Karamsad, Anand, Gujarat 388325 India; 2 Department of Neonatology, Pramukhswami Medical College, Bhaikaka University, Karamsad, Anand, Gujarat India; 3 Department of Central Research Services, Pramukhswami Medical College, Bhaikaka University, Karamsad, Anand, Gujarat India

**Keywords:** Health care, Medical research

## Abstract

Neonatal resuscitation training can change outcomes of neonatal mortality due to perinatal asphyxia. Recently, in 2021, the advanced NRP course material was changed, and for Basic NRP, a hybrid course was introduced in India. We assessed the instructor’s feedback to improve the conduct of the IAP NNF NRP Program as well as get their perception of the effectiveness, usefulness, and pitfalls of the new hybrid Basic NRP course (offline + online). A cross-sectional survey was conducted amongst instructors across India with current status with IAP NRP FGM Office. The data were exported to a Microsoft Excel Spreadsheet. STATA 14.2 was used for descriptive [Frequency (percent) analysis. 827 basic and 221 advanced NRP instructors responded. Bag and mask ventilation was identified as the most important step in basic 468 (56.6%) and advanced 147 (66.5%) courses. In the basic NRP, almost two third (71.0%) participants believe that it is challenging to conduct a case scenario for bag and mask ventilation, whereas, in the advanced course, intubation 116 (52.5%) was considered the most difficult step to teach and medication 80(36.2%) followed by intubation 62(28.1%) are the most difficult steps to conduct case scenario. 725(87.7%) reported that it would be easy to explain them in an offline course after completion of an online course. Most of the instructors were satisfied with the course structure, material, overall quality of the workshop, and support from the IAP NRP office. Constructive suggestions were obtained from the instructors for improvement of the course.

## Introduction

Neonatal mortality is high in lower and middle-income countries^1^ Perinatal asphyxia contributes to a significant amount of neonatal mortality^2^. Newborns that fail to initiate respiration can be assisted during the transition by providing effective assisted ventilation^3^. The Neonatal resuscitation program(NRP) was initiated in 1987 to address the need for an educational program focusing on the initial management of newborns immediately after the birth of the baby^4^. It offers a comprehensive and systematic training program that teaches skills such as psychomotor and teamwork in addition to changing the evidence in this area. The program has been adopted in 130 countries worldwide using contextual changes. Hence having healthcare personnel available to resuscitate neonates should be the norm in health systems that work towards reducing neonatal mortality. They must also be proficient in the knowledge, skills, and attitudes to deliver effective ventilation as per the latest recommendation^5^.

National Neonatology Forum (NNF) began training pediatricians and nurses in the Neonatal resuscitation program from 1987. However, the organization’s focus was on advanced neonatal resuscitation courses involving skills such as intubation and vascular access. There were limited resources (both manikins and instructors), and hence the number of healthcare personnel being trained was less. In the 1990s, the government of India began to focus on newborn care and launched various programs such as CSSM (Child Survival and Safe Motherhood), RCH (Reproductive and Child Health), etc., which culminated in a four-day course called Facility Based Newborn Care(FBNC) in 2011. This program has one day dedicated to the training of Neonatal Resuscitation, but this FBNC program was offered to only nurses and doctors in SNCUs (Special Newborn Care Unit). Thus, most nurses and doctors across India had limited opportunities to learn newborn resuscitation, often taught unsupervised and uncertified during in-service training. In the latter half of the first decade of the twenty-first century, there was an increasing realization that it was unnecessary to teach advanced neonatal resuscitation to everyone and ensure skills until Bag and Mask Ventilation would suffice for most newborns across the globe. Based on this understanding, the Government of India launched the Navajat Shishu Suraksha Karyakram (NSSK) in September 2009. This included Basic Newborn resuscitation, which included ventilation but not chest compression (on Day1), and essential newborn care that included breastfeeding, kangaroo mother care, prevention of hypothermia, and hand hygiene (on Day 2) as a two-day training course. The American Academy of Pediatrics (AAP) also launched the Helping Babies Breath program, which had similar course content as Day1 of NSSK in 2010 but a different teaching methodology^6^. The Indian Academy of Pediatrics (IAP) partnered with the Government of India to do NSSK training for Pediatricians across many states in India of NSSK and used the same resource material to train healthcare workers in the private sector. In 2011, IAP and NNF established a partnership to ensure that they work together in Neonatal Resuscitation. The neonatal resuscitation collaboration was named as IAP NNF NRP FGM (First Golden Minute Project), with a separate website and office to run the program with National, Zonal and Academic coordinators. This project aims to have one person trained in neonatal resuscitation at every delivery^7^. Subsequently, the resuscitation algorithm has been changed three times as per ILCOR (International Liaison Committee on Resuscitation) guidelines. IAP has used the AAP Textbook of Neonatal Resuscitation for its advanced courses while using the 2009 resource material of NSSK for Basic neonatal resuscitation courses. NNF has been involved in conducting only Advanced NRP courses. In 2014, NNF launched its own Neonatal Resuscitation India textbook, which was used as course material for the advanced NRP courses^8^.

In November 2020, ILCOR, AHA (American Hospital Association), and other organizations published newer guidelines. Consequently, the AAP released the newer version of the Textbook of Neonatal Resuscitation in June 2021. The IAP began utilizing this 8th edition for its training in October 2021. Thus in 2021, both the Basic NRP and the Advance NRP course material were changed. The government of India also changed the basic NRP algorithm in November 2020, and the NSSK resource material was changed. In view of the prevailing covid pandemic, to ensure that the changed course is disseminated faster, a hybrid course for Basic NRP was worked upon by the IAP NRP FGM academic group and reviewed at a meeting of highly experienced teachers in Mumbai in February 2021. This included videos of various sections of basic NRP that the participant must pass through to generate a pass certificate allowing him to attend a physical course nearby. After the release of the eighth edition, a decision to increase advanced NRP instructors was taken by the IAP NNF NRP FGM Core Committee. It was decided to use the eighth edition to teach interested members. As the number of covid cases decreased, from September 2021, many Advanced and Basic TOT (Training of Trainers) were conducted all over India till May 2022 to prepare more instructors with the aim of training more and more healthcare workers involved in newborn care.

In view of the changed curriculum, the conduct of courses, the structure, and the rollout of the Hybrid Basic NRP program, it was decided to assess the instructor’s feedback to improve the conduct of the IAP NNF NRP Program. The feedback would involve perceived effectiveness, pitfalls, and difference from only offline courses. Additionally, since many Basic NRP Instructors were Advanced NRP Instructors, we also utilized this opportunity to ask them about these courses.

## Results

A total of 827 Basic NRP instructors responded to the survey out of 2221 Instructors (37.23%), while 221 Advanced NRP Instructors responded to the advanced NRP survey out of 409 (54.03%). Table 1 shows the participants’ gender, experience, designation, and the year they completed their basic/advanced NRP Instructor training.

**Table 1:** Background/baseline characteristics of the study population.

Question	Basic	Advanced
	Frequency (%) N = 827	Frequency (%) N = 221
Age		
25–35 years	100(12.1)	37(16.7)
36–45 years	338(40.9)	105(47.5)
46–55 years	239(28.9)	56(25.3)
> 55 years	150(18.1)	23(10.4)
Gender		
Male	506(61.2)	139(62.9)
Female	321(38.8)	82(37.1)
Post-MD/DCH (Doctor of Medicine/ Diploma in Child Health) Post-graduation work experience in the year		
< = 5 years	36(4.4)	12(5.4)
6–10 years	159(19.2)	60(27.1)
11–15 years	185(22.4)	50(22.6)
16–20 years	154(18.6)	39(17.6)
21–30 years	195(23.6)	47(21.3)
> 30 years	98(11.9)	13(5.9)
Designation		
Senior resident	20(2.4)	6(2.7)
Tutor	23(2.8)	1(0.5)
Assistant professor	123(14.9)	51(23.1)
Associate professor	92(11.1)	39(17.6)
Professor	152(18.4)	33(14.9)
Other	417(50.4)	91(41.2)
Which year did you take your basic/advanced NRP Instructor training?		
Before 2000	17(2.1)	0
2000–2004	20(2.4)	1(0.5)
2005–2010	127(15.4)	17(7.7)
2011–2015	252(30.5)	29(13.1)
2016–2020	216(26.1)	60(27.1)
In 2021	195(23.6)	79(35.8)
In 2022	0	35(15.8)

In Advanced NRP instructors, Others constitute 41.2% which include Private Paediatricians, Obstetricians, Anaesthetist, Nurses etc. while in Basic NRP instructors, 50.4% are others, in which apart from above all, other health care workers are also instructors.

According to instructors, bag and mask ventilation is the most important step in basic 468 (56.6%) and advanced 147 (66.5%) courses. In the basic course, half of the instructors believe Bag and Mask Ventilation 415 (50.2%) is the most difficult step to teach, and almost two-thirds (71.0%) believe that it is challenging to conduct a case scenario for bag and mask ventilation, whereas, in the advanced course, intubation 116 (52.5%) was considered the most difficult step to teach and medication 80(36.2%) followed by intubation 62(28.1%) are the most difficult steps to conduct case scenario as shown in Table 2. Basic 721(87.2%) and advanced 188(85.1%) instructors believe they have the knowledge and skills to develop standardized scenarios that are both challenging and appropriate for the learner’s or provider’s level. Two-thirds of instructors in the basic 625 (75.6%) and advanced 152 (68.8%) courses said they have the knowledge and skills to teach neonatal resuscitation using low and high-fidelity simulators. In both basic 708 (85.6%) and advanced NRP 192(86.9%) reported that they get enough support from the IAP NRP office to conduct the course.

**Table 2:** Experience and knowledge of instructors.

Question	Basic	Advanced
	Frequency(%)N = 827	Frequency (%) N = 221
Number of basic/advanced NRP courses conducted as an instructor		
1–3	261(31.6)	92(41.6)
4–5	97(11.7)	27(12.2)
6–10	117(14.2)	16(7.2)
11–15	85(10.3)	14(6.3)
16–20	38(4.6)	5(2.3)
21–25	34(4.1)	3(1.4)
26–30	15(1.8)	1(0.5)
> 30	69(8.3)	6(2.7)
Not a single one	111(13.4)	57(25.8)
Most important step		
Bag & mask ventilation	468(56.6)	147(66.5)
Chest compression	NA	10(4.5)
Initial steps	264(31.9)	27(12.2)
Intubation	NA	30(13.6)
Routine care	95(11.5)	7(3.2)
Step most difficult to teach?		
Bag & mask ventilation	415(50.2)	59(26.7)
Chest compression	NA	19(8.6)
Initial steps	62(7.5)	8(3.6)
Intubation	NA	116(52.5)
Medication	NA	19(8.6)
Routine care	18(2.2)	0
None	332(40.1)	0
Difficult to conduct a case scenario?		
Bag & mask ventilation	587(71.0)	26(11.8)
Chest compression	NA	41(18.6)
Initial steps	145(17.5)	8(3.6)
Intubation	NA	62(28.1)
Medication	NA	80(36.2)
Routine care	95(11.5)	4(1.8)
Do you feel you have knowledge and skills to develop standardized_ Scenarios that are challenging & appropriate for the level of learner or provider?		
Maybe	88(10.6)	27(12.2)
No	18(2.2)	6(2.7)
Yes	721(87.2)	188(85.1)
Do you have knowledge & skills to use low & high-fidelity simulator to teach neonatal resuscitation?		
Maybe	112(13.5)	49(22.2)
No	90(10.9)	20(9.0)
Yes	625(75.6)	152(68.8)
Do you get enough support from IAP NRP office to conduct the course?		
Maybe	100(12.1)	25(11.3)
No	19(2.3)	4(1.8)
Yes	708(85.6)	192(86.9)

Some of the instructors have yet to participate as faculty in any NRP course. In advanced NRP. Out of 57, 50(87.71%) had undergone instructor courses in 2021 and 2022. In basic NRP, Out of 111, 63(56.75%) had undergone instructor courses in 2020 and 2021. The rest 48 (43.24%), had done the instructor course previously, but they had yet to do a single course as faculty. Among those 36 (75.0%) were from others. We believe those who had undergone the instructor course recently did not get an opportunity to become faculty.

More than half of the Basic NRP instructors, 477 (57.7%), reported a notable change in participant understanding following the hybrid course, and 725(87.7%) reported that it would be easy to explain them in an offline course if they had already completed the online course as shown in Table 3.

**Table 3:** Opinions regarding the new hybrid basic NRP course.

**Basic**	Frequency (%) N = 827
How many courses have you conducted after the implementation of the new hybrid course (online + offline) for basic NRP?	
0	290(35.1)
1	202(24.4)
2	110(13.3)
3	94(11.4)
4	35(4.2)
5	30(3.6)
6–10	50(6.1)
> 10	16(1.9)
Do you feel, Is there a significant change in the understanding of participants after the hybrid course?	
Maybe	246(29.7)
No	104(12.6)
Yes	477(57.7)
Do you feel it would be easy to explain them in the offline course when they have already done the online course?	
Maybe	78(9.4)
No	24(2.9)
Yes	725(87.7)

In a simulation scenario, more than half of the advanced NRP instructors use a laptop 125(56.6%), 82 (37.1%) use a mobile device, and the rest use a television 14(6.3%). Advanced NRP instructors 194 (87.8%) believe that the timings of each session are appropriate, while 5 (2.3%) believe that the time is too long and 22 (10.1%) believe that the time is too short. Nearly one-fourth of advanced NRP instructors, 49 (22.2%), said they needed more debriefing training, and 91 (41.2) said that they needed more simulation training as shown in Table 4.

**Table 4:** Opinions of Advanced NRP instructors regarding simulation and debriefing.

Advanced	Frequency (%) N = 221
What device was used for debriefing	
Laptop	125(56.6)
Mobile	82(37.1)
TV	14(6.3)
Time for each teaching session	
Appropriate	194(87.8)
Too long	5(2.2)
Too short	22(10.0)
Need for additional training in simulation	
Maybe	61(27.6)
No	69(31.2)
Yes	91(41.2)
Need for additional training in debriefing	
Maybe	63(28.5)
No	109(49.3)
Yes	49(22.2)
Received training in simulation (apart from NRP) N = 221	
No	97(43.9)
Yes	124(56.1)

Ending section.

We had also collected the data regarding state wise neonatal mortality rate, number of courses conducted in a year in each state of India (April 2021–March 2022) and state wise number of instructors. Pearson correlation coefficient of the number of NRP Courses and instructors was (r = 0.75), which is indicating that number of courses are largely dependent on number of instructors.

### Analysis of qualitative responses

#### Opinions regarding support from the IAP NRP office

Most instructors’ opinions were positive regarding support from the IAP NRP office. They said that the NRP office is doing an excellent job by providing facilities with all requirements, ample support, and adequate financial aid for Advanced NRP. In addition, the NRP Office staff is cooperative and provides quick solutions to problems on the phone or through email.

Basic NRP courses are well planned, with a good set of videos on each step, which will help the trainee refresh when necessary. The instructors have asked for more videos by Dr. Vikas Goyal for Advanced NRP steps, showing the resuscitation on the abdomen, breast crawl in normal and cesarean delivery, skin-to-skin contact, delayed cord clamping, and videos regarding registration of the course. The majority of instructors have a pleasant experience in conducting the hybrid course.

Instructors felt the need for the coordinated prompt delivery of the NRP kit with manikins and the provision of manikins personally to the instructor or district level with the involvement of local IAP. Instructors requested high fidelity manikins; more attractive pamphlets for conducting case scenarios, books, and videos in the local language, a uniform algorithm for basic and advanced NRP, regular updates and amendments for their and participants updates, more local instructors, rotation of instructors for the course, so everyone gets a chance, more budget including re-numeration for faculties as expenses are increasing day by day.

There are a few good suggestions for conducting more Basic NRP programs for paramedical staff in remote areas, and Basic NRP sessions for nurses and Nurses should lead nursing students.

Advanced NRP instructors asked for T piece resuscitator for demonstration and good manikins for intubation skills and lubricants. They felt that the quality control processes need to be taught to maintain manikins, and more training was needed for debriefing. In addition, there should be a faster dispatch of certificates.

#### Opinions about overall workshop qualities

Instructors opined about the overall workshop qualities. As per their opinions, NRP is a very well organized, particularly excellent quality workshop with a five-star rating in terms of training method, providing excellent hands-on experience. In addition, it adds much value to individual skills.

The recently introduced hybrid Basic NRP course is excellent, and it has become easier to teach skills offline, which is what most instructors feel. So the online followed by offline course is an excellent step to improve quality.

NRP training should be done in a small group, providing more time for the workshop, especially bag and mask ventilation. It would be good to ensure that all participants have hands-on practice with more case scenarios and post-test assessments after a few months to see retentiveness. There should be at least one senior instructor in the course who plans online meeting of all instructors before a few days and ensure uniformity among instructors for coordination and knowledge sharing, which senior faculties should supervise. It must be compulsory for the instructor to conduct a course at least once per year to increase the number of courses.

They gave specific suggestions for basic NRP such as IAP NRP should target govt hospital. The main problem is the shortage of manpower and lack of motivation. There is difficulty in referring the patients and transporting them, and they should focus on in states like Bihar, especially in north Bihar, Jharkhand, and Odisha. They should train obstetricians, government hospital doctors, and peripheral health care workers to have separate training for paramedics, giving them more time, and tests should be in the local language. Nurses should be taught a full equipment checklist of advanced NRP in the basic course as they might be working in a tertiary care center where they also have to prepare the equipment for advanced resuscitation. The ratio of participant to instructor should be reduced from 8:1 to 6:1 so that each participant will get a chance of hands-on practice on all skill sessions and make a small separate manual for basic NRP, including simulation, Briefing, and debriefing in the course with more time for debriefing.

Manikins transport has many issues like damage during transport, courier charges, time-consuming, and late deliveries. There is a shortage of manikins with many courses going on simultaneously all over the country. Few items from the kit may also be lost while transferring from one place to another, so it would be more convenient if it is provided to the local IAP branch; hence organizing the course and getting manikins will be much easier. Intubation without lubricants damages the manikins after repeated attempts to learn intubation.

To improve the quality of training, quality improvement strategies should be incorporated into the course, following a single course book and giving more time for simulation-based training-case scenario along with strict post-test evaluation of participants. Workshop timings should be increased, which can be done by making it hybrid by doing initial sessions online one day prior and hands-on with simulation for one day.

## Discussion

Eight hundred twenty-seven basic and 221 advanced NRP instructors responded. Most of the instructors were satisfied with the course structure, material, overall quality of the workshop, and support from the IAP NRP office. Constructive suggestions were obtained from the instructors for improvement of the course.

Currently, India’s neonatal mortality rate is 24.9/1000 live births^9^. The target for India is to reach a single-digit by 2030. Having at least one trained person to attend a delivery call is essential to achieving that goal^7^. There has been a reduction in childhood mortality due to various interventions in previous decades. We expect new and sustained initiatives like the NRP program and interval modifications in course structure after getting instructor and participant’s feedback will help in decreasing neonatal mortality. More resources should be allocated to ensure close surveillance of newborn infants immediately after birth^10–12^. Thus, in peripheral health centers, more emphasis should be given to these essential services.

In India, the Neonatal Mortality Rate (NMR) rate is almost double the Sustainable Development Goal target, with more than half of neonatal deaths occurring in only few states like Bihar, Chattisgarh, Uttar Pradesh, Uttarakhand etc.^9^. It was found in the study by Vail B et al. that various structural, logistical, and cultural barriers affect all aspects of immediate neonatal care and resuscitation in Bihar. Hence, the approach to this state must be different for it to achieve improvement in NMR^13^. Unfortunately, few advanced or Basic courses are being conducted in Bihar despite funding from the IAP NNF NRP FGM Program. So there is a need to focus on these states , preparing more instructors locally and encouraging them to do more and more courses in these states to train more health care workers.

The general doctors, midwives, and nurses in small rural areas made it clear that neonatal resuscitation programs needed to be practical, short, and approachable^6,14^. Hence the program of basic neonatal resuscitation to train workers involved in newborn care in the periphery is useful. Also, a simplified separate basic NRP algorithm greatly benefits this category of healthcare workers. However, when faced with a simpler basic NRP algorithm, advanced neonatal practitioners such as our instructors find it unwieldy as they often face complicated resuscitation scenarios. That’s why both algorithms are prepared as per need.

Every 5 years, International Liaison Committee on Resuscitation (ILCOR) publishes a consensus on cardiopulmonary resuscitation. The latest update on neonatal resuscitation guidelines was published in 2020^15^. Significant changes have been made in each update, and every time a guideline is changed, the instructors of each country must review the contents of the revised guideline to understand the changes made in the guideline^16^.

Ongoing skills practice, frequent retesting after the course, and refresher training are needed to maintain neonatal resuscitation skills. It helps to improve the course’s quality, and in this way, India can achieve its target^17^. Frequent, short, simulation-based training can hasten and maintain newborn ventilation skills in a multidisciplinary delivery unit staff in a high-resource setting^18^.

Instructors demanded high-fidelity manikins for the training program, but no difference was found in the outcome of randomized control trials of high-fidelity vs. low-fidelity simulation. There was no significant difference in the “improvement” between both the groups with respect to the written exam (*p* = 0.38) or Mega code assessment (*p* = 0.92)^19,20^. Further, the post-test and 3-month scores were comparable for the skills as well as content components, suggesting that the skills were retained in 3 months with an opportunity for self-learning^19,20^. We have recently carried out a similar type of study to compare high versus low-fidelity manikins for NRP training, which we will publish very soon. We also got a similar result as there were no significant differences in the outcome of high versus low-fidelity manikins.

The COVID (Corona Virus Disease) pandemic has had a lot of impact on medical education throughout the world. Newer approaches to traditional face-to-face courses, including various online formats, have resulted in instruction modifications to acquire knowledge and skill. mATLS (Mobile Advanced Trauma Life Support) offers an online didactic (knowledge) instruction platform, followed by face-to-face skills rotation and evaluation, thus reducing face-to-face interaction. It was found that mATLS delivery scores were as high or higher on the post-test than those who received only face-to-face instruction. So the mATLS may be able to replace in-person lectures of ATLS courses ^21^. Different videos on neonatal resuscitation are very much helpful in teaching learners effectively in a proper way as a result obtained in a study by et al. that application of the HybridLab® training technique, which consists of an e-learning platform followed by hands-on simulation, practical skill retention was better and drop in the skill performance was significantly less over a period of time^22^. We also found the same result for our recently introduced hybrid Basic NRP course in this study. So for advanced NRP also, we can increase workshop timings to provide sufficient time to practice the skills for which we can make it hybrid, which helps in better understanding to the participants like in basic NRP with added advantages of being more cost-effective and convenient.

Jordache R et al. studied the effect of a team sports briefing model on the neonatal resuscitation team. They found that the briefing-debriefing process could be transferred and beneficial to the performance of the NICU (Newborn Intensive Care Unit) team^23^. More emphasis should be given to it by giving additional training and more time for briefing-debriefing sessions in their instructor training.

Better clinical outcomes require dedication to quality improvement (QI). Providers who are devoted to quality improvement decide their goals, measure outcomes, identify gaps, and make changes that improve care^7^. Coordination and sharing should be uniform among instructors. Life support course instructors make fewer errors due to the retention of skills by teaching these skills at least twice a year^24^. Hence at least one senior experienced instructor should be there in each program. It must be compulsory for the instructor to conduct a course at least once per year to retain their teaching skills. Instructors should also need to be well‐acquainted with updated knowledge and innovative teaching methods, and organizations should promote valid recertification programs and support^25^. Ongoing development of NRP instructors has been accomplished via online sources like video clips, webinar, NRP current changes seminars in national conferences which is similar to processes followed by American Academy of Pediatrics^26^.

Instructors should follow standard guidelines and algorithms to maintain uniformity and give sufficient practice time. This is attempted by having an instructor meeting at the start of every course and, in recent times, having a zoom meeting of the instructors in the week before so that there is uniformity. Instructors should be experts with good psychomotor skills and competent enough to determine the errors in learners’ performance^27^. An instructor has to ensure that students achieve a high level of mastery during a training event and then several sessions to provide recent updates in a timely manner. Instructors should provide education as per the learner’s scope and practice environment and proper feedback regarding their performance. They must assess the learner participant’s cognitive, technical, and behavioral skills competency by validating the tool. Innovative educational strategies can be disseminated via gamified learning, social media, blogs and podcasts, and crowdsourcing. Neither the survey nor the responses from the instructors dwelled upon these aspects of instructor training or conduct of the course. Hence it would be necessary that in future instructor courses, the training should discuss the theory and practice of instruction and facilitation to improve the learning outcomes of the courses conducted. It may be prudent to involve experienced educators during the conduct of the courses as, currently, the instructors are mainly focused on skill transfer and less on the modality of teaching to improve learning.

The NRP office needs to make SOPs (Standard Operating Procedures) regarding the maintenance of manikins which should be an additional skill taught to all the instructors and participants and included in the NRP manual. This would improve the instructors’ confidence and increase their sense of ownership.

Since the research design is a cross-sectional one-time survey of instructors, it has all the limitations inherent in the study design, such as reporting bias, survey after a short duration of experiences, recall bias, varying situations, and participant composition, which might influence their opinions.

The current survey shows that the instructors are well-clued into the goals of the IAP NNF NRP FGM program and have given important feedback, which will ensure that the program improves in the future. A survey of the participants of these courses is currently being conducted and will also be helpful to get the perspective of the end-users.

## Materials and Methodology

### Setting

This cross-sectional survey was conducted amongst Advanced and Basic NRP instructors from all over India over a period of 5 months, from February 2022 to June 2022.

### Data collection instrument

Separate semi-structured questionnaires were prepared for Advanced NRP instructors and Basic NRP instructors. The questionnaire was based on the training they received, their experience in conducting the course, difficulties faced while conducting it, their opinions regarding high vs low fidelity simulation and debriefing. A high-fidelity healthcare simulation is any scenario reproducing an actual patient scenario to a high level of realism, including physical, environmental, psychological and other components.A low-fidelity simulation may lack several components that make the scenario feel more like the real world. Questionnaire consisted of two open-ended questions like support received from the IAP NRP office and suggestions to improve the course’s overall quality. In addition to this, we have taken basic NRP instructor’s feedback regarding effectiveness and added advantages of hybrid (online followed by offline) course over traditional offline course only.

The questionnaires were created in English for both. Pilot testing of the questionnaires was done prior to the actual survey on 30 instructors, and as a result, two question options were changed or added. So a pre-tested structured questionnaire was used to collect data from the instructors. Consensual validity was ensured after the necessary modifications and corrections were made. There were 21 questions in the Basic NRP instructor survey and 22 in the Advanced NRP instructor survey.

### Data collection

The Google forms were developed separately for both surveys and their appropriate links were shared with all Advanced and basic NRP instructors through email and social media platforms. The survey was administered via email and WhatsApp groups of Instructors and Individual messages via WhatsApp.

**Figure 1:**
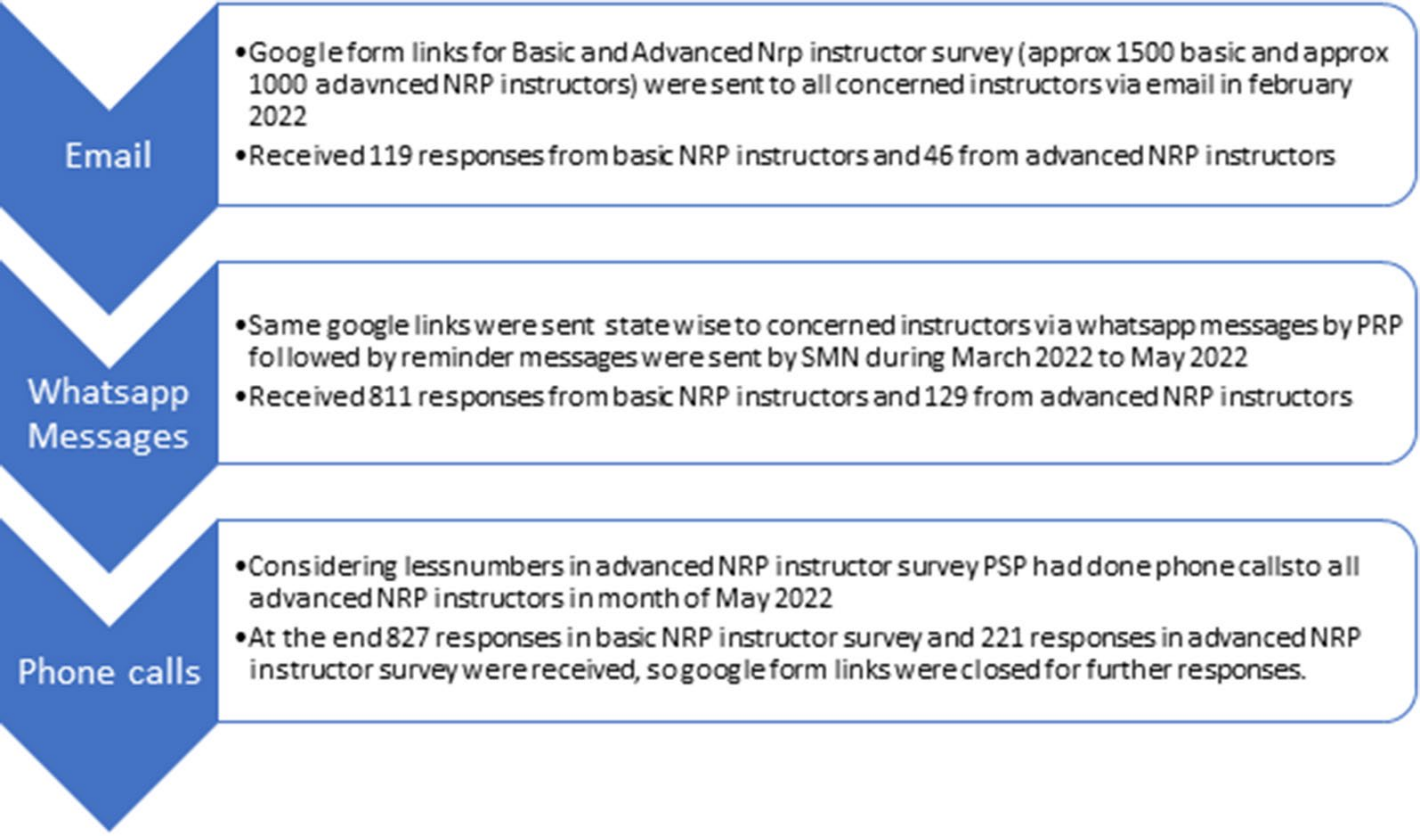
Recruitment process of participants of survey.

The recruitment process is summarized in Fig. 1.

#### Plan of statistical analysis

The data was exported from Google Forms to a Microsoft Excel Spreadsheet. STATA (Statistical Software for Data Science) 14.2 was used for analysis. Descriptive Statistics [Frequency (%)] were used to depict baseline characteristics and analysis. Pearson correlation coefficient (r) was calculated to find a linear relationship (correlation) between the number of basic NRP instructors and the number of Basic NRP courses conducted by instructors. The recruitment process to ensure more instructors filled out the survey is detailed in Fig. 1.

### Ethics

Institutional research committee 2 of Bhaikaka University(BU), Karamsad, Anand, Gujarat, approved the proposal via letter number IEC (Institutional Ethical Committee) /BU/2021/EX 46/313. All the participants gave informed consent to participate in this study. All methods were performed in accordance with relevant guidelines/regulations.

## Data Availability

The datasets generated and/or analyzed during the current study are not publicly available due to the researcher’s hesitation but are available from the corresponding author on reasonable request.
